# Coronavirus disease 2019 and gastrointestinal disorders in children

**DOI:** 10.1177/17562848231177612

**Published:** 2023-06-05

**Authors:** Anna Röckert Tjernberg, Petter Malmborg, Karl Mårild

**Affiliations:** Department of Pediatrics, Kalmar County Hospital, Region Kalmar County, Kalmar S-391 85, Sweden; Sachs’ Children and Youth Hospital, Södersjukhuset, Stockholm, Sweden; Department of Clinical Science and Education, Södersjukhuset, Karolinska Institutet, Stockholm, Sweden; Department of Medicine Solna, Division of Clinical Epidemiology, Karolinska Institutet, Stockholm, Sweden; Department of Pediatrics, Queen Silvia Children’s Hospital, Gothenburg, Sweden; Department of Pediatrics, Institute of Clinical Science, University of Gothenburg, Gothenburg, Sweden

**Keywords:** celiac disease, COVID-19, functional gastrointestinal disorder, inflammatory bowel disease

## Abstract

During the past 3 years, the coronavirus disease 2019 (COVID-19) pandemic has had a great impact on people all over the world. However, it has become evident that disease manifestations and severity differ across age groups. Most children have a milder disease course than adults but possibly more pronounced gastrointestinal (GI) symptoms. Given the child’s developing immune system, the impact of COVID-19 on disease development may differ compared to adults. This study reviews the potential bi-directional relationship between COVID-19 and GI diseases in children, focusing on common pediatric conditions such as functional GI disorders (FGID), celiac disease (CeD), and inflammatory bowel disease (IBD). Children with GI diseases, in general, and CeD and IBD, in particular, do not seem to have an increased risk of severe COVID-19, including risks of hospitalization, critical care need, and death. While infections are considered candidate environmental factors in both CeD and IBD pathogenesis, and specific infectious agents are known triggers for FGID, there is still not sufficient evidence to implicate COVID-19 in the development of either of these diseases. However, given the scarcity of data and the possible latency period between environmental triggers and disease development, future investigations in this field are warranted.

## Introduction

In December 2019, a novel coronavirus, later named severe acute respiratory syndrome coronavirus 2 (SARS-CoV-2), emerged and spread rapidly throughout the world.^1–6^ In March 2020, the World Health Organization (WHO) declared the coronavirus disease 2019 (COVID-19) pandemic, and as of January 27, 2023, over 752 million confirmed cases have been reported worldwide.^5,7^

SARS-CoV-2, a single-stranded RNA virus, commonly causes fever, cough, sore throat, runny nose, loss of smell or taste, and headache.^1,8^ A more severe disease course may result in pneumonia or respiratory and multiorgan failure.^1,8^ By January 27, 2023, the WHO dashboard reported 6,804,491 deaths from COVID-19, mainly in older adults, with a very low risk of death in children and younger people.^7^ In contrast to infected adults, most children and adolescents have only experienced mild disease symptoms.^7,9–12^

Although considered a respiratory disease, children and adults with COVID-19 frequently report gastrointestinal (GI) complaints (e.g. diarrhea, nausea, vomiting, and abdominal pain).^13–16^ Similarly, GI manifestations are prominent features of the multisystem inflammatory syndrome in children (MIS-C).^17,18^ MIS-C is a severe hyperinflammatory condition that can follow a COVID-19 infection in children and often requires aggressive treatment.^17,19^

Because SARS-CoV-2 can infect most parts of the GI tract,^20^ there has been a concern that the virus may influence the incidence of GI diseases and their disease course (or vice versa), such that patients with GI diseases may have an increased susceptibility to and risk of severe COVID-19. These questions were also raised for children often susceptible to respiratory viral infections and where viral encounters may imprint on their developing immune system.^21^

This study reviews evidence of potential bi-directional relationships between COVID-19 and pediatric GI diseases. While hepatic and pancreatic manifestations have been described in children with COVID-19,^14,16,22–24^ this review focuses on more common digestive disorders in children, such as functional GI disorders (FGIDs), celiac disease (CeD), and inflammatory bowel disease (IBD). We also review data on changed diagnostic work-up and management of children with GI diseases during and after the pandemic.

## COVID-19 and the gastrointestinal tract

COVID-19 usually causes respiratory symptoms in the upper and lower airways, as well as fever, headache, myalgia, and loss of smell and taste.^1–3,8^ However, GI complaints have also become common, especially in the pediatric population.^15,20,25^

SARS-CoV-2 can affect the GI system in several ways. The viral spike protein binds to membrane receptors, mediating a fusion between the virus and the membrane. Research has demonstrated that angiotensin-converting enzyme 2 (ACE2) receptor and transmembrane serine protease 2 (TMPRSS2) are crucial in this process. The ACE2 receptor and the TMPRSS2 are co-expressed not only in the lungs but also in the esophageal, gastric, and small and large intestinal mucosa, making the entire GI tract highly susceptible to SARS-CoV-2 infection.^13,14,20,26–28^ Of note, it seems that the expression of these proteins varies across ages. With increasing age, ACE2 and TMPRSS2 expressions decrease in the GI tract while increasing in the lungs.^29^ This finding may partly explain the noticeably milder disease course in children than in adults and perhaps age-related differences in COVID-19 symptomatology overall.

Once the virus has entered the cell, new virions are synthesized and assembled. Afterward, they are released into the GI tract, causing direct damage to the enterocyte and viral shedding in the stool.^13,26,30,31^ Fecal excretion of SARS-CoV-2 is common.^14,26^ In an early study from China Hua *et al.* detected SARS-CoV-2 RNA in feces in 32/35 (91%) infected children, with viral shedding persisting up to 70 days.^31^ This finding was confirmed in a 2022 meta-analysis reporting a pooled prevalence of fecal SARS-CoV-2 RNA in almost 9 of 10 infected children and 7 of 10 children who had turned negative in the respiratory specimen.^30^

Viral damage of the enterocytes may increase intestinal permeability and release of inflammatory mediators (e.g., interleukin 2 and tumor necrosis factor-alpha). These cytokines may induce GI symptoms and systemic inflammation.^13,20^ Furthermore, SARS-CoV-2 can alter the intestinal microbiota.^32^ Through immunological crosstalk between the gut and lungs (the gut-lung axis), a changing gut microbiome may also occur when the GI tract is not directly infected by the virus.^13,33^

### Gastrointestinal manifestations

Soon into the pandemic, a Chinese study reported clinical features of 651 hospitalized patients with COVID-19 (average age 46 years), with 11% having at least one GI symptom (nausea, vomiting, or diarrhea).^34^ Since then, similar or even higher rates of GI complaints have been reported in adult patients with COVID-19. A North American study including 1992 patients across 36 centers reported GI symptoms in up to 53% of the participants, with diarrhea being most frequently observed.^25^

GI complaints may be more common in children with COVID-19 than in adults,^10,15,35^ although comparisons are difficult given the heterogeneity of compared study populations. In contrast to adults, children with COVID-19 usually have a mild disease course that rarely requires medical attention.^11,12^ Whether the study cohorts constitute inpatients, outpatients, or household contacts will probably significantly impact the resolution and weighting of reported symptoms.

The Chinese Pediatric Novel Coronavirus Study Team assessed children who had been in contact with individuals with known or suspected COVID-19 in early 2020.^36^ Of 1391 tested children, 171 were SARS-CoV-2 positive (median age 6.7 years). Almost 16% of infected children were asymptomatic, but 9% reported diarrhea and 6% vomiting.^36^ The Peds COVID-19 Registry, a US study of >6000 hospitalized children^37^ reported vomiting in 13% of the cohort, abdominal pain in 10%, and diarrhea in 11% of infected children. Several reviews and meta-analyses have reported diarrhea in 2-33%, nausea or vomiting in 5–35%, and abdominal pain in 4–35%.^14,16,38^ Still, individual studies have reported a higher frequency of GI complaints, including GI symptoms in >50% of children in a North American multicenter study of severe pediatric COVID-19.^25^ These high numbers are in analogy with other viral infections (e.g., influenza); where children more frequently report GI symptoms than adults.^39^

### Multisystem inflammatory syndrome in children

Multisystem inflammatory syndrome in children (MIS-C) has proven to be one of the most serious COVID-19 manifestations in children and adolescents.^40^ MIS-C is a hyperinflammatory syndrome with multiorgan dysfunction seen in individuals <21 years of age with a recent history of COVID-19.^41^ Next to fever (mandatory criteria), GI symptoms are among the most predominant clinical manifestations of MIS-C, demonstrated in >90% of the patients.^17,18,41–48^ While diarrhea and vomiting seem to be the most common symptoms of MIS-C, abdominal pain, described in one-third of the affected children, is the most noticeable symptom, and often severe enough to warrant radiology or even surgery.^43,48,49^ This GI predominance of MIS-C differs from Kawasaki’s disease, a distinct infectious-related hyperinflammatory syndrome with usually no or only mild GI symptoms.^50–52^ Initially, MIS-C was associated with a significant mortality rate, particularly in low- and middle-income countries.^53,54^ However, with timely recognition of the condition and the development of improved anti-inflammatory treatment protocols, the morbidity and mortality rates of MIS-C have been reversed.^19^ Likewise, when patients are provided anti-inflammatory treatment, GI symptoms of MIS-C often resolve within a few weeks.^17,43,52^

## Celiac disease

Celiac disease (CeD) is a chronic autoimmune enteropathy precipitated by gluten intake in genetically predisposed individuals.^55^ The only available treatment for CeD is a strict gluten-free diet (GFD).^56^ In children, the prevalence of CeD is approximately 1% worldwide and possibly higher in Nordic countries.^56,57^ Human leukocyte antigen haplotype DQ2 or DQ8 is necessary but insufficient for CeD development.^55,58^ Besides gluten, other environmental factors contribute to CeD. Although the timing and nature of such environmental factors are largely unknown,^55,58^ multiple studies have acknowledged the implication of infections in disease pathogenesis.^59–64^ Conversely, CeD patients may also be susceptible to infectious diseases.^65–68^

### Celiac disease and risk of COVID-19

Over the past decades, CeD has consistently been associated with an increased risk of sepsis and infections with encapsulated bacteria (e.g., invasive pneumococcal disease and pneumonia).^66,68–71^ These risks likely relate to the higher prevalence of functional hyposplenism and splenic atrophy in patients with CeD.^72^ However, also increased intestinal permeability,^73,74^ altered intestinal microbiota,^75,76^ and impaired nutritional status^77,78^ may predispose celiac patients to infections. In this connection, pediatric and adult celiac patients have been linked to an increased risk of nonencapsulated serious infections,^65^ including a twofold risk of severe influenza.^79^ Although the susceptibility to serious infections seems to persist after introducing a GFD, the risk is likely somewhat higher in untreated CeD.^65^

Children with CeD do not seem to have an increased risk of symptomatic COVID-19. A population-based Swedish cohort study of 4490 patients with childhood-onset CeD observed no hospital admissions due to COVID-19 between February and July 2020 (and only one COVID-19-related hospitalization in 21,736 general population comparators).^80^ Similar null findings have been reported from Italy^81^ and a large international study in which pediatric and adult CeD patients had no significantly increased risk of COVID-19 [odds ratio 1.18 for laboratory-confirmed COVID-19, 95% confidence interval (CI) = 0.75–1.84].^82^ Additional international data from the Surveillance Epidemiology Under Research Exclusion for Celiac Disease (SECURE-CELIAC) Registry, collected between March 31, 2020, and February 23, 2021, have also confirmed hospitalization and fatality rates for children and adults to be similar in CeD compared to the general population.^83^ Also adult studies have unequivocally shown a similar incidence of COVID-19 in CeD as in the general population.^84–86^ Likewise, a large US study (TriNETX) found no significantly increased risk of hospital admission, critical care, or death from COVID-19.^87^

### COVID-19 and risk of subsequent celiac disease

Numerous viruses have been suggested to be involved in the development of CeD.^63^ In prospective studies, intestinal viruses, including rotavirus, enterovirus, and parechovirus infections, have been linked to an increased risk of CeD or CeD autoimmunity (i.e., CeD serology positivity).^59,63,88–90^ Moreover, several respiratory viral infections (e.g., influenza^60^ and respiratory syncytial virus^61^) have been associated with later CeD.^63,91,92^

The role of viruses in CeD pathogenesis remains to be elucidated. Still, they might contribute to the immunological loss of gluten tolerance through, for example, bystander activation or by increasing gut permeability, enabling epithelial translocation of gluten, or through increased expression of CeD-specific tissue transglutaminase.^64,93–96^ Both are critical elements in the pathogenesis of CeD. Likewise, induction of proinflammatory interferons from viral infections, including SARS-CoV-2, has been hypothesized to predispose to CeD.^97^

Data on COVID-19 and the subsequent risk of CeD are so far limited. A recent study from Türkiye reported a threefold increase in the annual number of CeD diagnoses in children during the pandemic (March 2020 through June 2021, *n* = 47) compared to the years before the pandemic (January 2008 through February 2020, *n* = 148).^98^ Of the 47 patients diagnosed during the pandemic, endoscopy was performed in 33 (70%). Although moderate-severe mucosal lesions (>Marsh 3a) were less common during the pandemic compared to the prepandemic years (42% *versus* 82%), the proportion of patients with classical CeD (e.g., diarrhea and poor growth) seemed to increase during the pandemic. Although this finding was not statistically significant, a potentially harmful delay in CeD diagnosis has been reported by other authors.^99^ Nevertheless, the interpretation of the results from the Turkish study^98^ is restricted by the retrospective nature of the data and the limited sample size. The potentially harmful delay in the diagnosis of CeD was reported in Italy.^99^ Italy had a very strict COVID-19 lockdown, particularly at the pandemic’s beginning. A recent Italian study demonstrated a 14% reduction in newly diagnosed CeD during the pandemic.^100^ The authors attributed this reduction to the diminished availability of health care services and possibly the fear of contracting COVID-19. Still, they could not entirely rule out the influence of other pathogenic factors or a natural fluctuation in CeD incidence.^100^ However, a prospective German study found that adult patients exposed to mild COVID-19 infection had persistent higher levels of CD-specific anti-tissue transglutaminase antibodies (compared to uninfected blood donors), also months after the infectious period, perhaps indicating a future rise in CeD incidence.^101^

### COVID-19 and adherence to a GFD

The COVID-19 pandemic has significantly impacted food supply chains, but supply disruptions have varied between countries. A US survey of 378 households of children with CeD reported decreased availability of gluten-free foods and an increase in intentional gluten intake.^102^ Difficulties in maintaining a strict GFD during the pandemic have also been reported in India.^103^ In contrast, reports from Italy and Türkiye have suggested unchanged GFD adherence levels of children with CeD.^104,105^

## IBD

IBD, consisting mainly of two conditions, Crohn’s disease (CD) and ulcerative colitis, is a chronic, complex, and costly immune-mediated inflammatory disease. Although most IBD patients are diagnosed in early adulthood, it is estimated that some 15% of all patients are diagnosed during childhood.^106^ The global incidence of childhood-onset IBD has increased significantly over the past few decades, with the highest IBD risks reported in Northern Europe and Canada.^107^ While genetic susceptibility plays a role in IBD,^108,109^ this rapid rise of childhood-onset IBD also suggests an important role of early environmental elements, where one candidate risk factor for IBD is infections.^110–112^ Moreover, a significant risk of disease- and treatment-associated infectious complications has also been observed in the modern immunomodulatory IBD treatment era.^113^

### IBD and risk of COVID-19

Children and adults with IBD have an increased risk of serious infections.^113,114^ Opportunistic, skin, and GI infections are among those that may pose problems for patients with IBD.^115^ There are numerous factors contributing to the infection susceptibility of IBD. First, vast immunological and genetic data have linked IBD development to an aberrant immune response (including the innate and adaptive immune systems), which alterations may also predispose to infections.^116^ Second, IBD-related bowel surgery and medical therapies for IBD, which suppress the immune system, are risk factors for severe infections.^117,118^ The highest risks of serious infections have been observed in IBD patients exposed to combinations of immunomodulatory drugs, especially those including corticosteroids.^115,119^

Early in the pandemic, concerns of an increased risk of COVID-19 and a more severe disease course in patients with IBD were raised. Part of that concern related to the immunosuppressive effect of many IBD-related drugs,^115^ but was also grounded in the fact that SARS-CoV-2 enters human cells via the ACE2 receptor, expressed on epithelial and endothelial cells of the GI tract,^20,28^ and up-regulated during inflammation. Hence, longitudinal studies have examined whether IBD patients are at risk of COVID-19 or severe COVID-19.

A recent comprehensive meta-analysis found no significant overall increased pooled relative risk (RR) of COVID-19 in IBD (RR 0.47, 95% CI 0.18–1.26) or across IBD subtypes.^120^ Instead, some data suggest a lower risk of COVID-19 in adult IBD patients compared to non-IBD patients,^121,122^ a phenomenon that might be related to ‘COVID shielding’, that is, patients with chronic conditions may take extra precautions to reduce the risk of infection.^123^ Several studies have reported fears and over-protection in children with IBD compared to their peers.^124–127^

Although a recent meta-analysis concluded no overall increased risk of COVID-19 hospitalization in IBD,^128^ individual adult studies have shown a higher risk of COVID-19 hospitalization in IBD patients compared to general population comparators, but with RR estimates varying considerably from an increase of 17–68%.^121,129^,^130^ Furthermore, in adults with IBD severe COVID-19 has mostly been observed in those of older age and with cardiovascular comorbidities.^131^

The age-related risk of COVID-19 hospitalization in IBD patients has been illustrated in the SECURE-IBD Registry. In this registry, where some 800 of more than 7000 reports concerned pediatric IBD, COVID-19 hospitalization rates ranged from 5% in 10 to 19-year-olds to 47% in those ⩾80 years.^132^ Also, reports to a European IBD database suggest a mild disease course of COVID-19 in children with IBD.^133^

Rates of severe COVID-19 (e.g. critical care admission or death) in IBD have been similar to those of the general population.^120,129,134^ Propensity-score matched data from US healthcare organizations showed adult IBD patients to have an incidence rate ratio of 0.85 (95% CI 0.69–1.06) for critical care needs after COVID-19 infection compared to the general population.^122^ The COVID-19 mortality rate appears similar in people with and without IBD.^120,122^ As expected, case fatality for inpatients with IBD is primarily confined to older patients and those having comorbidities. In contrast, few deaths have been reported in pediatric IBD patients. The mortality rate of children <19 years in the SECURE-IBD Registry is 0%.^132^

Evidence in adults with IBD suggests that corticosteroids, but not biologicals, were associated with adverse outcomes to COVID-19.^122,135,136^ Pooled estimates from a recent meta-analysis linked corticosteroid use with a nearly twofold increase in RR for hospitalization and a threefold risk of critical care need or death.^120^ These results have prompted expert consensus recommendations to perform individual risk-benefit assessments of COVID-19 severity and IBD activity and consider gradual tapering of systemic corticosteroids and switch to budesonide in IBD patients who test positive for SARS-CoV-2.^137^

Finally, besides case reports,^137,138^ neither pediatric IBD nor its treatment seems linked to the COVID-19-associated hyperinflammatory syndrome MIS-C.^139^

### COVID-19 and risk of subsequent IBD

Childhood infections have been suggested to impact the risk of IBD development, possibly mediated through perturbations of the gut microbiome.^140^ Infections are also important determinants for the maturation of the immune system and maintenance of mucosal barrier integrity.^141^ Various forms of gastroenteritis have been consistently shown to increase the risk of IBD.^142^ However, in early childhood also, respiratory infections have been associated with up to a three times higher risk of pediatric IBD.^143^ We also know that respiratory infections, the predominant infectious subtype of young children worldwide, usually cause GI manifestations in early life^39^ and influence the intestinal microbiome.^33^

A US study with data on 587 pediatric IBD cases showed an increased incidence of pediatric IBD diagnoses during the COVID-19 pandemic compared to the expected number based on historic time trend data.^144^ In contrast, the most extensive study of some 4300 IBD patients reported a lower risk of incident IBD following SARS-CoV-2 compared to a propensity-score matched cohort who tested negative for SARS-CoV-2 (RR 0.63; 95% CI: 0.58–0.70).^122^ The lower IBD incidence post-COVID remained essentially unchanged when restricted to IBD diagnosed >6 months after testing for COVID-19.^122^ The same study showed that IBD patients with COVID-19 were 1.3 times more likely to have a flare (defined by IBD-related hospitalization) at a 3-month follow-up than those without prior COVID-19 (6.77% *versus* 5.08%, *p* < 0.01; RR 1.33, 95% CI 1.18–1.51).^122^ Still, another large adult IBD study reported no significant impact of COVID-19 on clinical IBD activity.^145^

### COVID-19 and adherence to IBD treatment

Patients with IBD have been reported to continue to adhere to their medication regimens during the COVID-19 pandemic.^124,125,127^

## FGID

FGIDs are common in children and consist of various disorders believed to be associated with the GI tract but without structural or biochemical abnormality findings at routine examinations. According to the Rome IV criteria from 2016, pediatric FGIDs could be divided into over 15 subdiagnoses. The most common subtypes are irritable bowel syndrome, functional abdominal pain, constipation, and dyspepsia.^146,147^ While the etiology of FGIDs is undetermined, several factors proposed to be relevant to its pathophysiology,^148^ including increased intestinal permeability, dysbiosis, and immune functioning, have also been linked to COVID-19 and other infections.

### COVID-19 and risk of subsequent functional gastrointestinal disorder

An increasingly recognized subset of patients develops FGID after a GI infection. This association has mainly been studied in adult patient cohorts with laboratory-confirmed gastroenteritis, though the results have also been confirmed in some cohorts of children with GI infection.^149–152^ In particular, bacterial enteritis (e.g., salmonella, campylobacter, and shigella) and parasite infections (e.g., giardia) seem to enhance the risk of FGID.^151,153,154^ Research on viral infections and postinfectious FGID in children is limited. A 2009 study examining rotavirus infection as a potential trigger for chronic GI complaints of children in the US and Italy found a higher, albeit not statistically significant, prevalence of functional abdominal pain after rotavirus infection compared to controls (16% *versus* 7%, *p* = 0.31).^155^

New-onset GI symptoms have been reported in adults who have recovered from COVID-19^156^ but have yet to be observed in recovering children.^157^ A recently published study from India found that 36/320 (11%) adults with COVID-19 developed FGID-like symptoms at a 1-month follow-up; symptoms remained in some 7% of the COVID-19 patients at 6 months of follow-up.^158^ Moderate to severe COVID-19 and the presence of GI symptoms at the time of infection were negative predictors for persistent long-term FGID-like symptoms.^158^

Long COVID (also known as postacute COVID syndrome) refers to the potential long-term effects of COVID-19, including fatigue, dyspnea, and cognitive problems that persist or return at least 3 months after the onset of COVID-19. While this condition has primarily been reported in adults, systematic reviews suggest that long COVID may also occur in children and adolescents.^159,160^ However, GI symptoms of long COVID are usually not predominant but may represent up to 2% of affected children.^160,161^

## COVID-19 and pediatric gastroenterology practices

Related to the generally milder disease course in children than in adults with COVID-19, pediatric health care, compared to adult care, has been less impacted by the pandemic. Still, routines to practice pediatric care, including pediatric gastroenterology, have been affected.^162^ For example, the pandemic necessitated a rapid adaptation to remote care delivery,^163,164^ and a rise in virtual care for chronic GI conditions (e.g. IBD^165^ and CeD^166^). While patient satisfaction with virtual care is typically reported to be high,^164^ and its use has been linked to reduced travel costs and school absences, the effectiveness of virtual GI clinics has not been thoroughly studied. Also, physical examination and growth monitoring, important features of clinical decision-making, are typically not part of remote care.

The pandemic has had repercussions on digestive endoscopy in adults,^145^ but even more so in children, where for safety and comfort concerns, endoscopy is recommended to be performed under general anesthesia or deep sedation.^167^ Because the pandemic consumed almost all anesthetic support on a worldwide scale, pediatric gastroenterologists had to adapt to a clinical praxis with a significantly reduced capacity to perform endoscopic procedures essential to the assessment, treatment, and care of children with many GI conditions (e.g., CeD, eosinophilic esophagitis, and IBD).^168^ To counter the effects of reduced endoscopy capacity, pediatric papers suggested temporary reductions in required immunoglobulin A anti-tissue transglutaminase antibody thresholds for nonbiopsy-verified CeD diagnoses.^169^ In addition, there has likely been an increase in nonendoscopic monitoring of IBD activity, such as fecal calprotectin testing. Still, the effects on care quality related to the modified pediatric endoscopy practice remain largely unknown. The restrictions associated with the COVID-19 pandemic have also had a negative impact on pediatric gastroenterology training programs in North America and Europe.^170,171^

Finally, the COVID-19 pandemic spurred a rapid set-up of studies and surveillance registers of different patient groups (e.g., CeD and IBD patients).^83,132^ While these registers have mainly focused on adults, their data include children and adolescents, which has enabled updated information on COVID-19 risks and real-time data to support management and treatment decisions.

## Comments

In summary, the risk of contracting and being seriously ill with COVID-19 seems comparable for children with CeD or IBD as for the general population.

Also, so far no firm evidence links COVID-19 with an increased risk of CeD, IBD, or FGID. Still, 3 years into the COVID-19 pandemic, its potential impact on CeD, IBD, and FGID has not been fully determined. Speculatively, there may be a long latency period between infectious exposures and the possible development of these diseases. Hence, given the ongoing intense spread of SARS-CoV-2 worldwide, the potential bi-directional association between COVID-19 and pediatric GI conditions needs further investigation. The pandemic might also have resulted in delayed GI diagnosis: families with children with GI complaints might have avoided hospitals for fear of contracting the virus.^124–127^ The reduced endoscopic capacity might have delayed the work-up of those symptoms.^145^

This review discusses the evidence of the development or exacerbation of pediatric GI conditions amidst COVID-19. However, we acknowledge that the COVID-19 pandemic has also carried a significant mental health burden on children globally,^172^ which may be even greater in children with chronic illness.^173^

The COVID-19 pandemic significantly impaired pediatric gastroenterology training programs and hampered our capacity to diagnose and follow children with GI complaints endoscopically. The pandemic’s effects on pediatric gastroenterology practices could last for years. On the other hand, concerted efforts and investments to combat delayed training and backlogs can likely limit these adverse effects.

## Declarations

**Ethics approval and consent to participate:** Not applicable.

**Consent for publication:** Not applicable.

**Availability of data and materials:** Not applicable.
